# Effect of PAC on the Behavior of Dynamic Membrane Bioreactor Filtration Layer Based on the Analysis of Mixed Liquid Properties and Model Fitting

**DOI:** 10.3390/membranes10120420

**Published:** 2020-12-14

**Authors:** Chunyan Huang, Hongju Liu, Shujuan Meng, Dawei Liang

**Affiliations:** School of Space and Environment, Beihang University, Beijing 100191, China; chunyanhuang@buaa.edu.cn (C.H.); liuhj@buaa.edu.cn (H.L.); mengsj@buaa.edu.cn (S.M.)

**Keywords:** DMBR, PN/PS in EPS, membrane fouling, the combined model

## Abstract

Recently, dynamic membrane bioreactor (DMBR) has gradually gained the interest of researchers for the development of membrane technology. In this paper, we set up parallel experiments to investigate the effect of powder activated carbon (PAC) on organic matter removal, transmembrane pressure, and filter cake layer characterization to make an overall performance assessment of DMBR. The results showed that DMBR has a good removal effect on organic matter removal, and with a chemical oxygen demand removal rate over 85%. Protein was found to be the main membrane fouling substance. Due to the electric double-layer effect, membrane fouling tended to be alleviated when the PN/PS value was low. Using a filtration model under constant current conditions, the filtration process through the cake layer was observed to be consistent with cake-intermediate model.

## 1. Introduction

Membrane bioreactor (MBR) has been widely used to treat various types of wastewater, include saline [1], municipal [2], industrial [3], dairy [4] and textile [5] wastewaters, due to its characteristic of thorough solid-liquid separation. The core of the MBR process is its porous membrane, which provides high quality reusable effluent [6]. MBRs also have some outstanding merits in terms of small footprints and capacity of resisting the impact of large change in influent quantity [7]. However, the membrane fouling in MBRs lead to their high operational costs and maintenance costs, and limit their further wide-spread usage. To solve the MBR membrane blocking problems, some scholars explore the use of alternative dynamic membrane bioreactor (DMBR) instead of traditional MBRs. DMBR belongs to a new category of MBRs [8], where the filter layer formed by suspended solid particles present in the filtered liquid is covered on a cheap, coarsely porous filter material, called secondary membrane [9]. The dynamic membrane uses traditional membrane fouling for water-sludge separation, and the microbial activity and proliferation shows an inseparable relationship with their metabolites [10]. The emergence of DMBR has huge potential as it might enable MBR technology to overcome its existing membrane blocking problems, while maintaining the characteristics of the MBR system to effectively retain biomass [11,12,13]. The DMBR tends to achieve high flux, high-quality permeability, complete biomass retention, low energy consumption and reduced membrane cleaning, compared with the microporous membrane materials used in MBR system. The support materials used in DMBR are large pore size materials that can be readily obtained at low price. Various studies have reported the use of filter materials such as nylon [14], terylene [15], stainless steels and polyester mesh [16] in DMBR. By using the method of material modification, copper foam has also been used as a filter material to achieve good results [17].

The filtration mechanism of DMBR is different from traditional membrane filtration. In DMBR, the dynamic membrane (DM) layer acts as the main source of filtration resistance after its formation [18]. Since the DM layer is a kind of surface sludge layer that can release smaller particles, the deposited organic material when gets more than the filtration capacity will block the membrane pores and cause membrane fouling issues in DMBR. It is reported that different foulant species may form aggregations through electrostatic attraction, hydrophobic interaction and hydrogen bonding interaction. These agglomerations can affect the interaction of foulants with other materials [19,20], extracellular polymer substances (EPS). Thus, it is significant to further investigate effects of EPS on membrane fouling as it has a greater impact on certain characteristics of activated sludge.

The DM layer occupies an indispensable position in the DMBR. The quality of the DM layer further affects the performance of the entire system. As DM is formed by the deposition of particles and colloids in the mixed liquid, the nature of the mixed liquid affects the filtration behavior of the DM layer. Therefore, the filterability and stability of the system can be improved by improving the mixed liquid to form a more permeable and stable DM layer. Previous studies have shown that adding powder activated carbon (PAC), flocculants, and adjuvant to the mixed liquor can achieve good result [21,22,23]. Due to the porosity and surface properties of PAC, it can not only improve the filterability and stability of the system by improving the mixed liquid, but can also provide a carrier for the growth of microorganisms, allowing microorganisms to grow, that can promote the removal of slowly, biodegradable organic matter [24].

To improve and render the property of DM layer to enhance wastewater treatment efficiency, in this study, we added PAC to the DMBR system in order to alleviate membrane fouling by increasing system porosity, improving permeability and enhancing system microbial activity, the reports on which are scarce.

Few studies have attempted to predict the formation of DMs through modeling [25]. In order to better evaluate the application of DMBR and allow an improved insight into the formation process of the cake layer, this study aims to (1) explain the formation and filtration performance of DMBR by supplementing it with PAC, (2) investigate the influence of PAC on the structure and stability of DMBR filter layer and membrane fouling substances, and (3) explore the filtering mechanism of dynamic membrane filtration layers by means of mathematical models. This study may provide information to further increase the knowledge and provide guidance for the application of DMBR system.

## 2. Materials and Methods

### 2.1. Operation of DMBR

Our equipment was a lab-scale 6.8 L DMBR and module material was PVC plates. The system used nylon with a pore size of 52 μm as the filter material and the total effective filtration area was 0.0114 m^2^. General characteristics of the dynamic membrane bioreactor are summarized in Table 1. The sludge used for inoculation was taken from the Tsinghua University’s Water Treatment Station (Beijing, China), and was used after being acclimated for two weeks. The initial experimental MLSS was 5 g·L^−1^ and the experiment was operated at 23 ± 2 °C. The filtration experiments were conducted at a constant flow (50 L·m^−2^·h^−1^) for 40 days with constant influent feeding. Three identical air generating devices were evenly placed at the bottom of the reactor to continuously generate oxygen required for microbial activity (1 L·min^−1^). The hydraulic retention time (HRT) was set to 8.5 h. A high-precision vacuum pressure gauge was installed on the pipeline between the water outlet and the reactor to measure the transmembrane pressure (TMP) change during the experimental operation. The experiment was parallelly divided into two groups, one group was operated without PAC, while the other was operated with a dose of 1 g·L^−1^ PAC. The particle size of PAC for the experiment was 44 μm and it was washed with deionized water before use. The other conditions were exactly the same. The DMBR was fed with synthetic municipal wastewater by peristaltic pumps (Longer Pump, BT100-2J, YZ1515x). The composition of the feeding is 0.169 g·L^−1^ glucose, 0.169 g·L^−1^ peptone, 0.063 g·L^−1^ (NH_4_)_2_SO_4_, 0.044 g·L^−1^ KH_2_PO_4_, 0.063 g·L^−1^ NaCl, 0.0233 g·L^−1^ CaCl_2_, and 0.094 g·L^−1^ NaHCO_3_, 0.094 g·L^−1^ MgSO_4_·7H_2_O, 0.0022 g·L^−1^ FeSO_4_·7H_2_O. During the experiment, a 10 mL supernatant sample was taken from the reactor daily for chemical oxygen demand and NH_3_-N analysis, while 50 mL was taken for the analysis of sludge properties every five days.

### 2.2. Analytical Methods

#### 2.2.1. Filtration Resistance Analysis

The filtration resistance (*R*) resistance was calculated according to Darcy’s formula:

(1) $$R=\frac{\Delta P}{\mu J}$$

Equation (1), where *R* is the filtration resistance of DMBR (m^−1^), Δ*P* is the transmembrane pressure (Pa), *μ* is the permeate viscosity (Pa·s), *J* is the effluent flux (m^3^·m^−2^·s^−1^).

#### 2.2.2. Extraction and Analysis of EPS, SMP (Soluble Microbial Products)

EPS is defined as a high-molecular weight polymer and a sticky substance derived from microorganisms. It is the main component of biofilms and is commonly found in cell lysates [26]. In addition to carbohydrates and proteins, EPS also includes other organic substances such as nucleic acid molecules [27]. In this study, EPS refers to the biopolymer substances extracted from the mixed solution, and SMPs are the soluble high molecular weight substances in the supernatant of activated sludge. SMPs were obtained by centrifuging 20 mL of the mixed solution at 3000 rpm for 10 min, which was then filtered through a 0.22 μm acetate cellulose membrane. EPS were extracted according to the method described previously [28]. Most existing studies suggest that EPS and SMP are mainly composed of polysaccharides and proteins which can be used to measure the content of SMP and EPS. Polysaccharides and proteins were determined by the phenol-sulfuric acid method [29] and the modified Lowry method [30], respectively.

#### 2.2.3. SEM Analysis

After the filtration, a small piece of nylon mesh used for filtration and containing the DM layer was cut flat with a scalpel, and then it was placed in a freeze dryer (Christ, Germany) for drying. Its morphology was observed with a field emission scanning electron microscope (FESEM) (ZEISS Sigma 500, German).

#### 2.2.4. Other Terms Analysis

Chemical oxygen demand (COD), ammonia, and biomass concentration were determined using the standard methods (APHA, 1998). To measure the Zeta potentials of mixed liquid sample, Malvern ZetaSizer Nano ZS was applied. The pH value of the mixed solution during the filtration process was measured using a pH meter (PE28, METTLER TOLEDO, Shanghai, China).

#### 2.2.5. Data Analysis

It is a good idea to analyze and understand the filtration behavior and DM formation mechanism with the help of models. Four classic models of Hermia’s general equations for describing filtered data under constant pressure filtration conditions were obtained as follows Equation (2).

(2) $$\frac{d^{2}t}{dV^{2}}=k\left(\frac{dt}{dV}\right)$$

where, *k* is a constant and *n* is the blocking index equal to 2, 1.5, 1, or 0 for complete blocking, standard blocking, intermediate blocking, and cake filtration, respectively.

**Figure 1:**
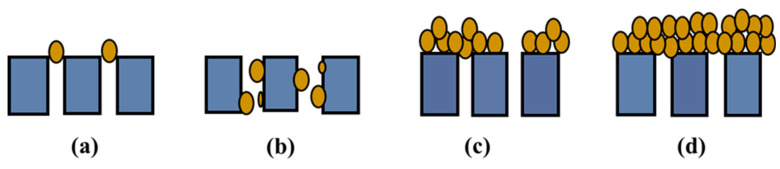
Illustration of the four classical filtration model: (**a**) complete blocking, (**b**) standard blocking, (**c**) intermediate blocking, and (**d**) cake filtration.

The four classical filtration models can help us further understand and evaluate the filtration capability and formation of DM during the operation of dynamic membrane bioreactor [31,32]. These are (a) complete blocking model, (b) standard blocking model, (c) intermediate blocking model and (d) cake filtration model, which are illustrated in Figure 1.

However, some researchers think that only one model is not enough to describe the actual filtering process under constant current state. Hence, the combined model was considered in this study. In this work, five forms of combined blocking models [33] considered for application are listed in Table 2.

## 3. Results

### 3.1. Filtration Performance

**Figure 2:**
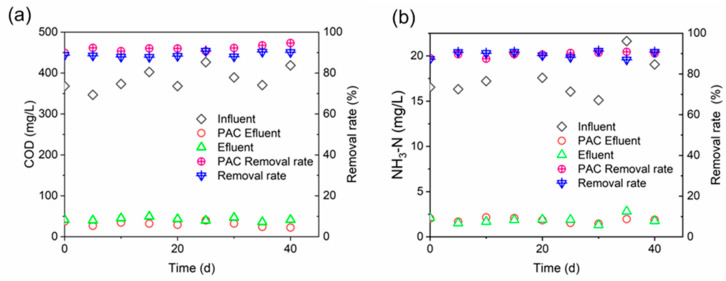
Performance of DMBR in synthetic wastewater treatment (**a**) COD removal; (**b**) NH_3_–N removal in the reactor.

As a carrier in DMBR, PAC can provide optimum conditions for microbial growth and form a stable biofilm ecosystem, which is conducive to achieve good growth and succession of microorganisms [34,35,36]. We tested the COD and NH_3_-H concentrations of the influent and effluent of C-DMBR (Efluent) and PAC-DMBR (PAC Efluent), and calculated the removal rate to evaluate the organic matter removal performance of the system. Figure 2 shows that DMBR has a fairly good performance in the removal of COD, and the COD removal rate for PAC-DMBR reaches up to 94.64%, which is better than 87.75% in DMBR without adding PAC. It shows that the addition of PAC can promote the degradation of organic matter, which can be attributed to the fact that PAC provides growth carriers for microorganisms, which promotes the interaction of microorganisms and PAC in activated sludge to form biological activated carbon. The development of biological activated carbon can increase the biodegradability of the system, such that the organic matter in the system can be adsorbed and completely degraded, which will help improve the system’s organic matter removal rate, and the adsorption of PAC can also promote the removal of organic matter [34,37]. At the same time, it was also observed that there was little difference in the removal rate of ammonia nitrogen between the two systems, it may be that the system has sufficient aeration and the short-cut nitrification and denitrification capabilities of the two systems are the same.

### 3.2. Membrane Resistance Analysis

**Figure 3:**
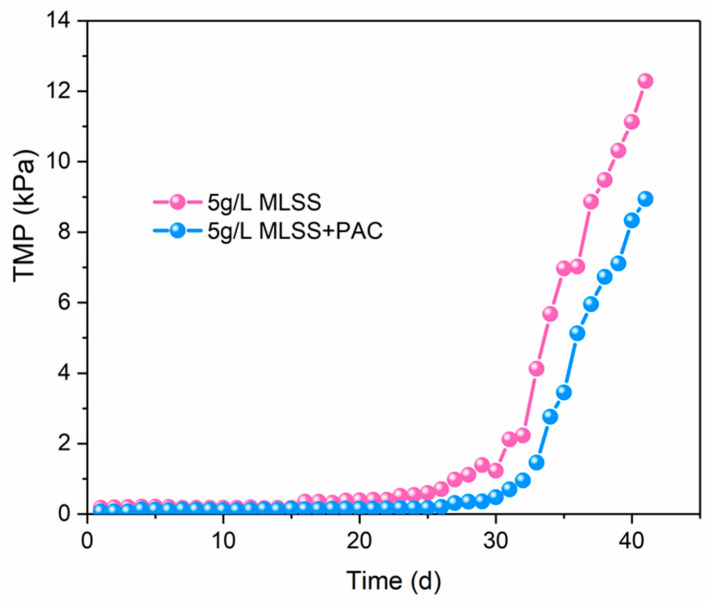
TMP profile at constant flux during operation in C-DMBR and PAC-DMBR.

Figure 3 describes that the trans-membrane pressure (TMP) of system was kept low and stable in both of the DMBRs, during the initial 30 days. After 30 days of stable operation of the reactor, the TMP increased up to 10 kPa with the increase in filtration time which indicates a threshold for the end of the first experiment cycle. This phenomenon has also been reported in many studies [38]. Obviously, compared to the DMBR without PAC (C-DMBR), filtration period was prolonged in PAC-DMBR, and a low TMP increase in PAC-DMBR has been observed during the experimental period, indicating that the addition of powdered activated carbon in the DMBR system can result in a good performance of the reactor by extending the filtration cycle and reducing the number of cleanings.

**Figure 4:**
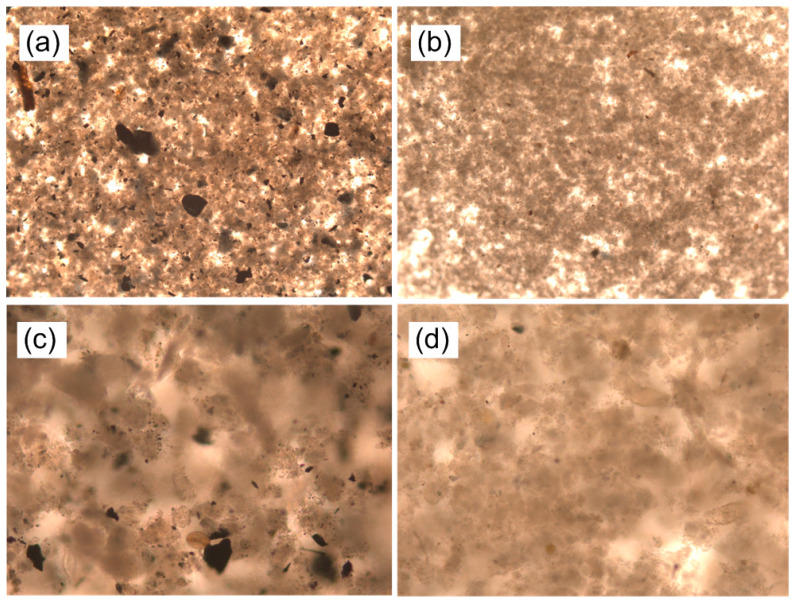
Microscopic examination of two systems: (**a**) 5×, (**c**) 10× of PAC-DMBR; (**b**) 5×, (**d**) 10× of C-DMBR.

Considering the nature of PAC, it is further believed that after adding PAC to the system, the original suspended sludge flocs are more likely to adsorb each other and aggregate to make larger flocs. In order to confirm this claim, we performed microscopic inspections on the two system mixtures, and the results are shown in Figure 4. The results indicate that the particles of the PAC-DMBR system are larger, and the microscopic examination results are consistent with our statement. The addition, PAC also prevented the colloidal material from being deposited in the membrane pores, and reduced their contact with each other, thereby improving the composition and permeability of the sludge cake layer [39]. Additionally, it was reported that the average TMP rising rate in MBR was 1.87 kPa/day, while it was only 0.27 kPa/day in DMBR, indicating that DMBR might be a promising application in wastewater treatment.

**Figure 5:**
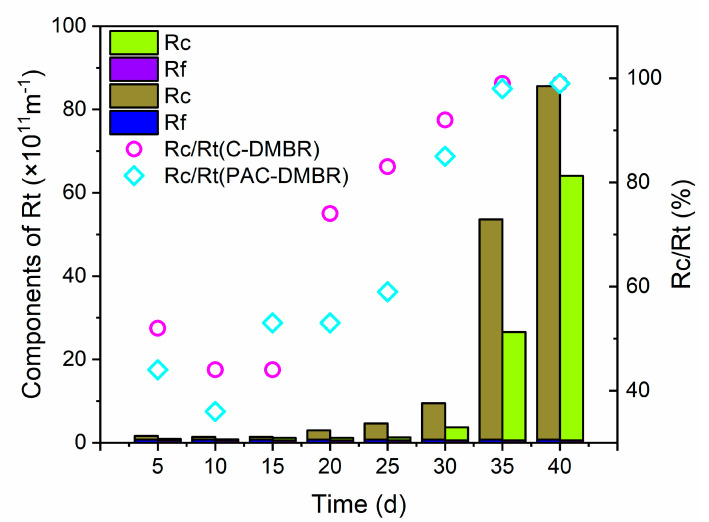
Resistances of two systems during operation time.

Generally, the total resistance (*R_t_*) is divided into three parts, namely the nylon mesh resistance (*R_m_*), pore blocking resistance (*R_f_*), and cake layer resistance (*R_c_*), which can be described by the following expressions [40]:

(8) $$R_{t}=R_{m}+R_{f}+R_{c}$$

where, *R_m_* is the intrinsic resistance of nylon module (m^−1^), which was measured via clean water filtration test. Thus, in this study, the nylon mesh resistance (*R_m_*) was ignored, as there was no reading displayed on the vacuum meter. Resistances of two systems during operation time are summarized and showed at Figure 5. From the results it can be confirmed that *R_t_* is mainly caused by the cake layer, and this conclusion is similar to the previous report [41]. Results also suggest that the filtration performance of cake layer can be improved by optimizing the coagulation and agglomeration characteristics of the mixed liquid containing PAC. From this, it can be assumed that the PAC in the cake layer can also resist the influence of filtration shears caused by hydraulic fluctuations in the filtration process and increase the stability of DM layer. However, further evidence is required to support this view which will the focus of our next work.

As it can be observed, the cake layer filtration resistance (*R_c_*) increased gradually from 1.616 × 10^11^ m^−1^ to 85.634 × 10^11^ m^−1^ in C-DMBR with time, while in PAC-DBMR it increased from 0.923 × 10^11^ m^−1^ to 64.090 × 10^11^ m^−1^ during the 40 days operation period, as shown in Figure 5. The same trend was observed with TMP variation. This further illustrates that the addition of PAC improves the permeability of the DM layer, as combined with the results of TMP analysis, the addition of PAC reduces both. In general, the total filtration resistance (*R_t_*) of DMBR is much lower than that of the traditional membrane bioreactors (10^12^–10^14^ m^−1^) [42], which demonstrates the advantage of DMBR in water treatment applications.

### 3.3. EPS and SMP Analysis of Samples

**Figure 6:**
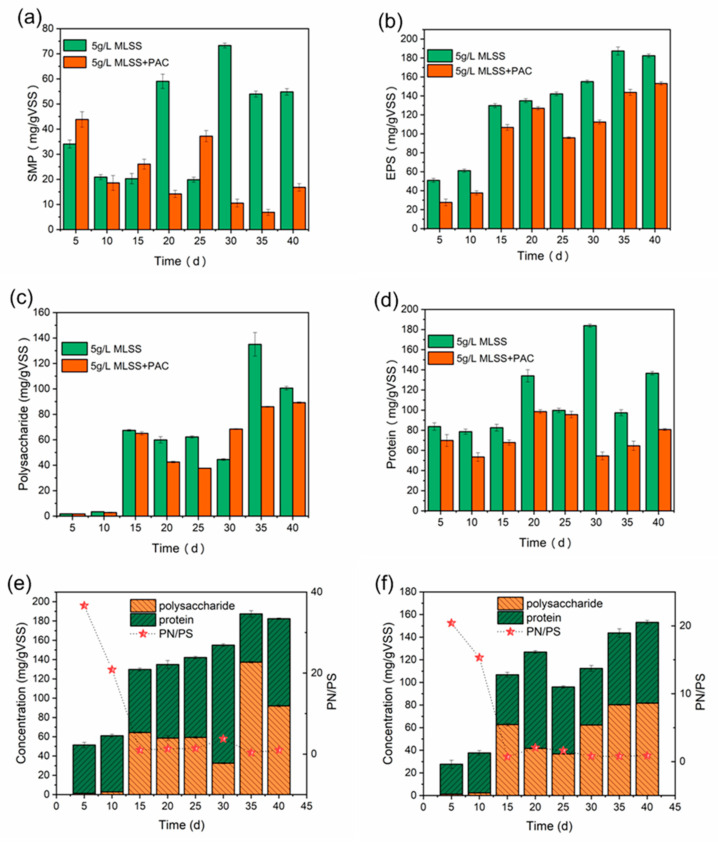
Contents and composition of (**a**) SMP, (**b**) EPS, (**c**) polysaccharide, (**d**) protein in sludge cake layer and (**e**,**f**) the proportion of polysaccharides and proteins in EPS of the sludge.

The content of EPS and SMP in sludge and its composition analysis is shown in Figure 6. As shown in Figure 6b, the content of EPS increased from 50.94 mg/g VSS to 187.36 mg/g VSS in C-DMBR, while in PAC-DMBR it increased from 27.67 mg/g VSS to 143.71 mg/g VSS, indicating that the addition of PAC can reduce the content of EPS, it may be that the adsorption of PAC enhanced the degradation of organic pollutants in the mixed solution. The content of polysaccharides and proteins in C-DMBR was higher than that in PAC-DMBR, as shown in Figure 6c,d. It has been observed that the protein/polysaccharide (PN/PS) ratio in PAC-DMBR decreased from 20.41 to 0.79, while 36.64 to 0.36 in C-DMBR, as shown in Figure 6e,f. Existing reports suggest that the value of PN/PS in EPS plays an important role for sludge flocculation, while the cake layer fouling is due to the hydrogen bonds, carboxylate and amino functional groups in polysaccharides and proteins which are related to the hydrophobicity and negative surface charge [43,44]. Therefore, the PN/PS value affects the properties of sludge flocs, and due to the double-layer compression in the sludge mixture, cross-linking between EPS and inorganic ions, and charge neutralization. The flocculation of sludge varies with the protein content and decreases with the increase of total EPS [45]. It is speculated that the low PN/PS value in EPS will help promote system sludge flocculation, which further shows that adding PAC can alleviate membrane fouling.

In addition, the average zeta potential of the mixed liquid for PAC-DMBR is lower than C-DMBR listed in Table 3. That is, the average zeta potential during PAC-DMBR operation is lower than that of C-DMBR, while two system mixtures remain neutral during operation. This implies that the sludge particles of PAC-DMBR can agglomerate into larger sludge floes, so that the cake layer of PAC-DMBR has higher porosity and better permeability. In this study, low PN/PS appear to be good for agglomeration and alleviation of membrane fouling.

### 3.4. SEM Analysis of Filtration Cake

**Figure 7:**
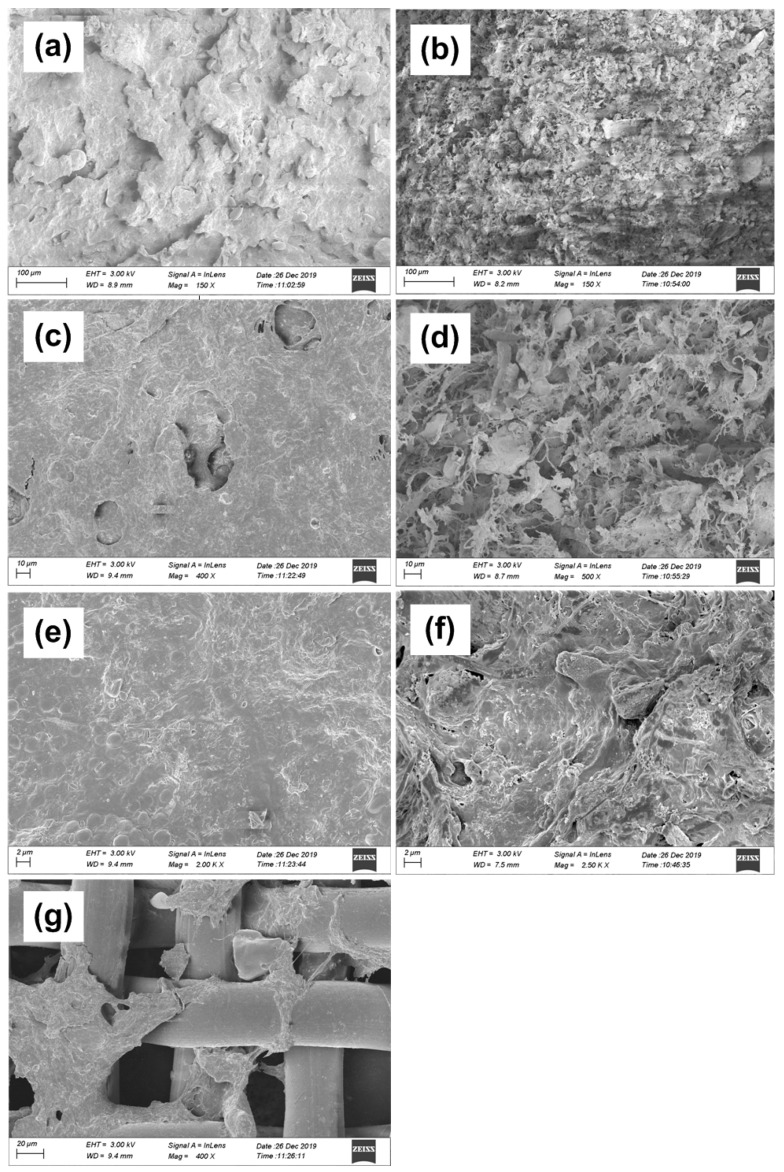
SEM images of the dynamic membrane samples of C-DMBR and PAC-DMBR (**a**,**c**,**e**) are cake layer for C-DMBR, (**b**,**d**,**f**) for PAC-DMBR, (**g**) the DM after the cake layer is scraped off.3.5. Model fitting of filtration cake.

To further study the DM layer morphology, the samples were analyzed by SEM images. Filter layers with different pore sizes can be observed from the images Figure 7a–f. Here, Figure 7a,c,e are images of the cake layer in C-DMBR, while Figure 7b,d,f are of PAC-DMBR. The results show that the surface of the cake layer is smoother, and almost covered by the gel layer, which increases the resistance to the permeation of liquid in C-DMBR (Figure 7c). While, the cake layer was observed to be loose and porous in case of PAC-DMBR (Figure 7d). It is obvious that the activated carbon and gel together form a larger structure where the skeleton participates in the agglomeration of the particles. The gel layer of PAC-DMBR, which was relatively loose, is beneficial for filtration, as shown in Figure 7e,f. Through the combined analysis of SEM images and particle size diagrams, it was concluded that the existence of PAC provides a base for larger particle formation, and at the same time, the activated carbon is used as the skeleton and the particles are cross-linked with colloids to form larger agglomerates. The filter layer structure has higher porosity and better permeability. It can be observed from Figure 7g that the structure after the cake layer was scraped off. It clearly indicated that that the viscous gel layer was stuck around the pores of the support material, and most of the gel did not enter the cavity. This echoes the curve fitting result. Thus, the effect of the internal pore blockage was weak.

It was conceived that the formation of the DMBR filtration layer should be similar to the process of fouling layer formation on the micro-filtration membrane. Accordingly, the formation of the cake layer can be understood by the means of filter cake filtration mechanism. This helps us better understand the membrane fouling process. We innovatively try to use the fitting results to explain the dynamic membrane fouling, and this work has not yet been reported.

**Figure 8:**
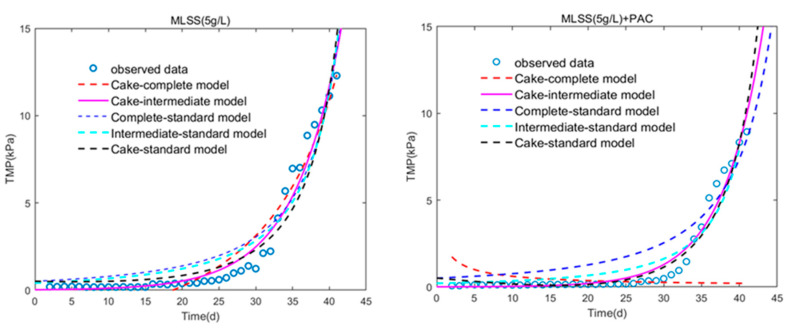
Curve fitting of the observed TMP data with the combined models.

The combined model was used to perform curve fitting analysis of the TMP behavior of the filtration experiment (Figure 8).

We can consult the parameter information from Table 4, and the result shows that the cake-intermediate model can better describe the filtration process, which is slightly different from the model fitting used in MBR in a previous research [46]. In other words, the entire filtration process of DMBR significantly involves the cake layer model and intermediate model. Thus, the formation and filtration mechanism of the cake layer in the DMBR filtration process can be described by the initial deposition of sludge particles and blocking of the membrane holes of the support material. The particles are then deposited on the top of the formed sludge to form a dense filterable filter layer. At the same time, the curve fitting analysis results of the five groups of combined models show that, except for the cake-intermediate model, the complete-standard model is closer to the filtering process than other combinations, followed by the intermediate-standard model. Considering the values of K_c_ and K_i_ present in the cake model, K_c_ plays a more important role as K_c_ > K_i_. It can be stated that the formation process of the cake layer is dominated by the filter layer formation and the intermediate blockage, and then finally develops into the cake layer to play the main role. This can also explain why the dynamic membrane filter layer is easy to fall off the surface of the support material. After physical cleaning, the filter material can be restored to the state that was before filtration, which is one of the most attractive advantages of dynamic membranes.

## 4. Conclusions

DMBR is a good alternative to MBR for its low consumption, easy to operate and good treatment performance. The results show that DMBR has a good organic matter removal effect and the removal rate can reach 94.64% when PAC is added. Considering that the polysaccharides are more easily biodegraded, protein is the main substance polluting the filter layer in this study, and the PN/PS value is related to sludge flocculated particles. The value of PN/PS in PAC-DMBR was less than in C-DMBR, indicating that the addition of PAC affects the protein and polysaccharide content of the mixed solution, which alleviates the membrane fouling process. The analysis of PSD and SEM showed that the addition of PAC made the mixed liquid particles larger, optimized the structure of the filter layer and affected the filtration performance. The model fitting showed that the combined model can well describe the filtration process of DMBR. The filtration behavior of DMBR can be described by cake-intermediate model, which shows that particle deposition and filtration in DMBR rarely affects the pores, but instead deposits on the surface of the membrane pores to form a filter layer causing the DMBR cleaning phase easier. This inspired us to optimize the filtration effect of the system by improving the properties of the mixed liquid forming the filter layer.

## Figures and Tables

**Table 1:** General information about the DMBR filter component.

Configuration	Plate Membrane
Material	Nylon mesh
Double-sided effective filtration area	0.0114 m^2^
Hydrophilicity	hydrophilic
Dacron mesh pore size	52 μm
fabric filter weight	110 (/cm)
Membrane module size	0.092 m × 0.062 m

**Table 2:** Combination of five models under constant flow.

Model	Equations		Fitted Parameters
Cake-complete	$\frac{P}{P_{0}}=\frac{1}{1-K_{b}t}\left(1-\frac{K_{c}J_{0}}{K_{b}}\ln\left(1-K_{b}t\right)\right)$	(3)	K_c_(s·m^−2^), K_b_(s^−1^)
Cake-intermediate	$\frac{P}{P_{0}}=\exp\left(K_{i}J_{0}t\right)\left(1+\frac{K_{c}J_{0}^{2}}{K_{i}}\left(\exp\left(K_{i}J_{0}t\right)-1\right)\right)$	(4)	K_c_(s·m^−2^), K_i_(m^−1^)
Complete-standard	$\frac{P}{P_{0}}=\frac{1}{\left(1-K_{b}t\right){\left(\left(1+K_{s}J_{0}/2K_{b}\right)\ln\left(1-K_{b}t\right)\right)}^{2}}$	(5)	K_b_(s^−1^), K_s_(m^−1^)
Intermediate- standard	$\frac{P}{P_{0}}=\frac{\exp\left(K_{i}J_{0}t\right)}{{\left(1-\left(K_{s}/2K_{i}\right)\left(\exp\left(K_{i}J_{o}t\right)-1\right)\right)}^{2}}$	(6)	K_i_(m^−1^), K_s_(m^−1^)
Cake- standard	$\frac{P}{P_{0}}=\left({\left(1-\frac{K_{S}J_{0}t}{2}\right)}^{-2}+K_{c}J_{0}^{2}t\right)$	(7)	K_c_(s·m^−2^), K_s_(m^−1^)

**Table 3:** The mean values of zeta and pH in the experiment.

Parameter	C-DMBR	PAC-DMBR
Zeta Potential	−16.1 ± 0.3	−11.9 ± 0.4
pH	7.17 ± 0.14	7.06 ± 0.11

**Table 4:** Linear parameters, correlation coefficients and model parameters for the five combined models using in this study.

Model	SSE	R-Squared	Fitting Parameters		
Cake-Complete Model	49.6568	0.9008	K_c_ K_b_	−0.7132 0.0168	s/m^2^ s^−1^
Cake-Intermediate Model	15.7609	0.9659	K_c_ K_i_	0.4198 0.1766	s/m^2^ m^−1^
Complete-Standard Model	44.6724	0.9033	K_b_ K_s_	−0.001707 0.005783	s^−1^ m^−1^
Intermediate-Standard Model	39.7477	0.9140	K_i_ K_s_	0.6329 0.4921	m^−1^ m^−1^
Cake-Standard Model	33.9338	0.9322	K_e_ K_s_	−0.001686 0.006901	s/m^2^ m^−1^

## Abbreviations

The following abbreviations are used in this manuscript:

MBR	Membrane Bioreactor
DMBR	Dynamic Membrane Bioreactor
HRT	Hydraulic Retention Time
DM	Dynamic Membrane
COD	Chemical Oxygen Demand
PS	Polysaccharide
EPS	Extracellular Polymer Substances
SMP	Soluble Microbial Products
PAC	Powder Activated Carbon
TMP	Transmembrane Pressure
PN	Protein
